# Draft Genome Sequence of *Cellulosilyticum* sp. I15G10I2, a Novel Bacterium Isolated from a Coal Seam Gas Water Treatment Pond

**DOI:** 10.1128/genomeA.01616-16

**Published:** 2017-02-16

**Authors:** Joseph Adelskov, Bharat K. C. Patel

**Affiliations:** Microbial Gene Research and Resources Facility, School of Natural Sciences, Griffith University, Brisbane, Queensland, Australia

## Abstract

*Cellulosilyticum* sp. strain I15G10I2 was isolated from a coal seam gas water treatment pond at the Spring Gully water treatment facility, Roma, Queensland, Australia. Analysis of the genome of 4,489,861 bp and G+C content of 35.23% revealed that strain I15G10I2 shared limited similarity to members of the genus *Cellulosilyticum*, family *Lachnospiraceae*.

## GENOME ANNOUNCEMENT

*Cellulosilyticum* sp. strain I15G10I2 was isolated from a coal seam gas (CSG) water treatment pond sample collected from the Spring Gully water treatment facility, Roma, Queensland, Australia. Strain I15G10I2 was isolated in a medium containing 0.1% NaCl, 0.1% NH_4_Cl, 0.03% H_2_KPO_4_, 0.01%, MgCl_2_.6H_2_O, 0.001% CaCl_2_.2H_2_O, 50 mM HEPES, 0.01% yeast extract, 0.02% sodium thioglycolate, 0.2% ammonium ferric iron citrate, and 4 mM 4-methoxy benzoate (p-anisic acid) anaerobically at 25°C following the method of Ogg et al. (1, 2). Strain I15G10I2, a strict anaerobe, grew optimally at 37°C and pH 8 and utilized glucose, fructose, xylose, arabinose, galactose, sucrose, starch, cellobiose, xylan, and dextrin as carbon sources. The 16S rRNA gene sequence (1,489 bp) revealed that the closest phylogenetic neighbors were *C. lentocellum* DSM 5427^T^ (93.71%) and *C. ruminicola* strain H1^T^ (92.85%), the only two taxonomically validated members of the genus *Cellulosilyticum* (3), family *Lachnospiraceae*, commonly associated with ruminants and human digestive tracts (4). Here, we present the draft genome sequence of isolate I15G10I2, a novel anaerobe from a CSG water treatment pond.

High-molecular-weight DNA of strain I15G10I2 was extracted using a modification of Marmur’s method (5) and submitted to the Australian Genomic Research Facility (AGRF) for TruSeq library preparation and sequencing on the Illumina MiSeq platform with specifications set for paired-end (PE) sequencing (2 × 250-bp read lengths). The sequencing of 718,395 PE reads (359,197,500 bp) were quality trimmed and filtered to 524,113 PE reads (258,522,023 bp) using Trimmomatic (6), and reads sharing an overlap of 30 bp with a maximum overlap difference of 10% were joined using fastq-join (7). The joined PE reads (179,454) and unjoined PE reads (344,659) were assembled using SPAdes version 3.5.0 (8) to produce a genome consisting of 30 contigs (*N*_50_ of 419,226 bp, coverage of 56.7×) with 4,489,861 bp and a G+C% content of 35.23. Prokka version 1.11 (9) annotation identified 4,170 protein-coding genes and 84 RNA genes. Phyla-AMPHORA (10) analysis of 56 universal genes extracted from 186 representative genomes of the family *Lachnospiraceae* confirmed that strain I15G10I2 was most closely related to members of the genus *Cellulosilyticum*, but ANIb and the Genome-to-Genome Distance Calculator (11) showed a weak relationship (averages of 72.93% and 19.6%, respectively), suggesting that it was a new species of the genus. RAST (12) analysis of strain I15G10I2 showed that 2,507 genes shared an identity of less than 50% (BLAST) to genes in the two *Cellulosilyticum* genomes combined, providing further evidence of its novelty.

### Accession number(s).

The annotated draft genome sequence of strain I15G10I2 was submitted to the European Nucleotide Archive under the project accession number PRJEB11913, sample accession number ERS1325239, and contig accession number FMMP0100000.

## References

[1] Ogg CD, Patel BK 2011 *Caloramator* *mitchellensis* sp. nov., a thermoanaerobe isolated from the geothermal waters of the Great Artesian Basin of Australia, and emended description of the genus *Caloramator*. Int J Syst Evol Microbiol 61:644–653. doi:10.1099/ijs.0.023655-0. 20400665

[2] Ogg CD, Spanevello MD, Patel BKC 2011 Exploring the ecology of thermophiles from Australia’s Great Artesian Basin during the genomic era, p. 61–97. *In* Satyanarayana T, Littlechild J, Kawarabayasi Y (ed.), Thermophilic microbes in environmental and Industrial biotechnology. Springer, Netherlands. doi:10.1007/978-94-007-5899-5.

[3] Cai S, Dong X 2010 *Cellulosilyticum* *ruminicola* gen. nov., sp. nov., isolated from the rumen of yak, and reclassification of *Clostridium lentocellum* as *Cellulosilyticum* *lentocellum* comb. nov. Int J Syst Evol Microbiol 60:845–849. doi:10.1099/ijs.0.014712-0. 19661493

[4] Meehan CJ, Beiko RG 2014 A phylogenomic view of ecological specialization in the *Lachnospiraceae*, a family of digestive tract-associated bacteria. Genome Biol Evol 6:703–713. doi:10.1093/gbe/evu050. 24625961 PMC3971600

[5] Kanso S, Patel BKC 2004 *Phenylobacterium* *lituiforme* sp. nov., a moderately thermophilic bacterium from a subsurface aquifer, and emended description of the genus *Phenylobacterium*. Int J Syst Evol Microbiol 54:2141–2146. doi:10.1099/ijs.0.63138-0. 15545448

[6] Bolger AM, Lohse M, Usadel B 2014 Trimmomatic: a flexible trimmer for Illumina sequence data. Bioinformatics 30:2114–2120. doi:10.1093/bioinformatics/btu170. 24695404 PMC4103590

[7] Aronesty E 2011 eu-utils: command-line tools for processing biological sequencing data. https://www.msi.umn.edu/sw/ea-utils.

[8] Bankevich A, Nurk S, Antipov D, Gurevich AA, Dvorkin M, Kulikov AS, Lesin VM, Nikolenko SI, Pham S, Prjibelski AD, Pyshkin AV, Sirotkin AV, Vyahhi N, Tesler G, Alekseyev MA, Pevzner PA 2012 SPAdes: a new genome assembly algorithm and its applications to single-cell sequencing. J Comput Biol 19:455–477. doi:10.1089/cmb.2012.0021. 22506599 PMC3342519

[9] Seemann T 2014 Prokka: rapid prokaryotic genome annotation. Bioinformatics 30:2068–2069. doi:10.1093/bioinformatics/btu153. 24642063

[10] Wang Z, Wu M 2013 A phylum-level bacterial phylogenetic marker database. Mol Biol Evol 30:1258–1262. doi:10.1093/molbev/mst059. 23519313

[11] Meier-Kolthoff JP, Auch AF, Klenk HP, Göker M 2013 Genome sequence-based species delimitation with confidence intervals and improved distance functions. BMC Bioinformatics 14:60. doi:10.1186/1471-2105-14-60. 23432962 PMC3665452

[12] Aziz RK, Bartels D, Best AA, DeJongh M, Disz T, Edwards RA, Formsma K, Gerdes S, Glass EM, Kubal M, Meyer F, Olsen GJ, Olson R, Osterman AL, Overbeek RA, McNeil LK, Paarmann D, Paczian T, Parrello B, Pusch GD, Reich C, Stevens R, Vassieva O, Vonstein V, Wilke A, Zagnitko O 2008 The RAST server: rapid annotations using subsystems technology. BMC Genomics 9:75. doi:10.1186/1471-2164-9-75. 18261238 PMC2265698

